# The ABC transporter gene family of *Daphnia pulex*

**DOI:** 10.1186/1471-2164-10-170

**Published:** 2009-04-21

**Authors:** Armin Sturm, Phil Cunningham, Michael Dean

**Affiliations:** 1 Institute of Aquaculture, University of Stirling, Stirling, FK9 4LA, UK; 2 Department of Biochemistry, King's College London, Franklin Wilkins Building, 150 Stamford Street, London, SE1 9NH, UK; 3 Laboratory of Genomic Diversity, National Cancer Institute, Frederick, MD 21702, USA

## Abstract

**Background:**

The large gene superfamily of ABC (ATP-binding cassette) transporters encodes membrane proteins involved in trafficking processes across biological membranes and further essential cell biological functions. ABC transporters are evolutionary ancient and involved in the biochemical defence against toxicants. We report here a genome-wide survey of ABC proteins of *Daphnia pulex*, providing for the first time information on ABC proteins in crustacea, a primarily aquatic arthropod subphylum of high ecological and economical importance.

**Results:**

We identified 64 ABC proteins in the *Daphnia *genome, which possesses members of all current ABC subfamilies A to H. To unravel phylogenetic relationships, ABC proteins of *Daphnia *were compared to those from yeast, worm, fruit fly and human. A high conservation of *Daphnia *of ABC transporters was observed for proteins involved in fundamental cellular processes, including the mitochondrial half transporters of the ABCB subfamily, which function in iron metabolism and transport of Fe/S protein precursors, and the members of subfamilies ABCD, ABCE and ABCF, which have roles in very long chain fatty acid transport, initiation of gene transcription and protein translation, respectively. A number of *Daphnia *proteins showed one-to-one orthologous relationships to *Drosophila *ABC proteins including the sulfonyl urea receptor (*SUR*), the ecdysone transporter *ET23*, and the eye pigment precursor transporter *scarlet*. As the fruit fly, *Daphnia *lacked homologues to the TAP protein, which plays a role in antigene processing, and the cystic fibrosis transmembrane conductance regulator (CFTR), which functions as a chloride channel. *Daphnia *showed two proteins homologous to MDR (multidrug resistance) P-glycoproteins (ABCB subfamily) and six proteins homologous to MRPs (multidrug resistance-associated proteins) (ABCC subfamily). However, lineage specific gene duplications in the ABCB and ABCC subfamilies complicated the inference of function. A particularly high number of gene duplications were observed in the ABCG and ABCH subfamilies, which have 23 and 15 members, respectively.

**Conclusion:**

The *in silico *characterisation of ABC transporters in the *Daphnia pulex *genome revealed that the complement of ABC transporters is as complex in crustaceans as that other metazoans. Not surprisingly, among currently available genomes, *Daphnia *ABC transporters most closely resemble those of the fruit fly, another arthropod.

## Background

ATP-binding cassette (ABC) proteins constitute one of the largest protein superfamilies and are present in all organisms from bacteria to human [1,2]. Prototypical ABC proteins are membrane-bound transporters coupling ATP hydrolysis to the translocation of substrates across biological membranes [3,4]. In addition to transporters, ABC proteins also comprise ion channels, regulators of ion channels, receptors, and proteins with roles in ribosome assembly and translation. The human genome has 48 genes encoding ABC proteins, of which 17 have been linked to hereditary diseases, including cystic fibrosis, adrenoleukodystrophy, Stargardt disease and disorders of cholesterol metabolism [2,5,6].

ABC proteins share a conserved domain architecture. A functional transporter requires the cooperation of two transmembrane domains (TMD) and two cytosolic nucleotide binding domains (NBDs, also called ATP-binding cassettes). Eukaryotic ABC proteins are either full transporters combining all required domains in one polypeptide (2 TMDs and 2 NBDs), or half-transporters consisting of 1 TMD and 1 NBD that need to form homo- or heterodimers to constitute a functional pump. According to their domain architecture and sequence, metazoan ABC transporters are divided into subfamilies, of which seven (A to G) exist in human [7]. An eighth subfamily (H) has been defined following the analysis of the *Drosophila melanogaster *genome [2]. The H subfamily is missing in mammals, but has one member in zebrafish [8]. The members of subfamilies E and F are not transporters and differ from other ABC proteins in that they possess two NBDs but lack TMDs. ABCE proteins are inhibitors of RNAse L and involved in the assembly of the preinitiation complex [9-11], while ABCF proteins have roles in ribosome assembly and protein translation [12,13].

The first eukaryotic ABC transporter discovered was the human (h) drug efflux transporter MDR (multidrug resistance) P-glycoprotein (hABCB1/MDR1), the name of which reflects that its expression in cancers can cause a decreased cellular drug accumulation (initially referred to as drug permeability, 'P'), resulting in the resistance of tumours against chemotherapy [14,15]. Subsequent studies have identified further ABC proteins that are drug efflux pumps and can cause MDR in cancers, including the multidrug resistance associated protein (hABCC1/MRP1) [16] and the breast cancer related protein (hABCG2/BCRP) [17]. Drug efflux transporters are found in ABC subfamilies B, C and G [18], and in normal tissues often show an apical expression in epithelia involved in excretion or forming boundaries of the body, reflecting their role in the biochemical defence against toxicants [19]. ABC drug efflux transporters have a wide phylogenetic distribution and are found in vertebrates as well as in deuterostome invertebrates (sea squirt *Ciona intestinalis *[8]; sea urchin *Strongylocentrotus purpuratus *[20]), protostome invertebrates (nematode *Caenorhabditis elegans *[21]; fruitfly *Drosophila melanogaster *[2]), protozoans and yeast [22,23]. Homologous proteins are also present in plants, though their roles in multidrug efflux have not been firmly established [24,25].

The planktic crustacean *Daphnia *is globally distributed and has central importance for the ecology of lakes and ponds. The currently accomplished sequencing of the *Daphnia pulex *genome will thus enhance research in disciplines which traditionally have made use of daphnids, e.g., ecology, physiology, toxicology, population genetics and behaviour. Moreover, studies with an evolutionary perspective are further expected to benefit, because *Daphnia *is not only the first crustacean, but also the first non-insect arthropod to have its genome sequence determined. The aim of this study was to provide a survey of the ABC transporter gene family of *Daphnia pulex*. A complete or close to complete list of ABC transporters in *Daphnia *will facilitate the identification of genes that play a role during the adaptation of *Daphnia *to environmental toxicants. Furthermore, ABC genes have been suggested as biochemical factors contributing to the phenomenon of resistance against chemotherapeutics in parasites [22,26] and insects [27,28]. In the aquaculture industry, parasitic crustaceans constitute a problem of considerable economic importance. The potential development of resistances against therapeutics used to control the crustacean parasite sea louse (*Lepeophtheirus salmonis*) in salmon farming is currently becoming a concern, and it has recently been proposed that ABC transporters in the sea louse could represent potential biochemical resistance factors to emamectin, a therapeutic used to control sea louse infestations in salmon [29,30]. At the same time, the sulfonylurea receptor has been suggested to represent the target for the chitin synthesis inhibitor diflubezuron [31], a compound also used to treat sea lice infections. Thus, ABC transporters might have relevance in crustaceans both as biochemical defence mechanisms against toxicants, and as targets of toxicity.

From an evolutionary perspective, the wide distribution of ABC transporters capable of drug efflux transport suggests they are of ancestral origin. However, ABC subfamilies containing drug transporters (B, C, and G) also comprise proteins with other functions. For instance, the arguably best known drug efflux pump hABCB1/MDR1 belongs to the B subfamily that also contains mammalian transporters of bile salts and phospholipids, and yeast transporters of pheromones. Because of the lack of clear orthologous relationships between ABC proteins of the nematode worm *Caenorhabditis elegans *and those of other genomes, it has been suggested that drug efflux pumps have evolved independently several times [21]. The annotation of ABC transporters in the *Daphnia *genome, provided by this work, represents an important resource for future biochemical, toxicological and physiological studies of ABC drug efflux transporters

## Results and discussion

To identify gene loci encoding ABC transporters, multiple tblastn searches were performed on the Dappu v1.1 draft genome sequence assembly (September, 2006) [32] using NBDs of different *Drosophila melanogaster *ABC proteins as queries (one search per subfamily). The most plausible gene model was selected among machine-generated models available at wFleaBase  and the JGI genome portal , and its NBDs extracted for phylogenetic analysis that further included NBDs of fruit fly and human ABC transporters. This resulted in a subfamily-specific clustering of NBDs, with N- and C-terminal NBDs of full transporters generally allocated to distinct sub-clusters (data not shown). Based on the clustering of NBDs, *Daphnia *transporters were assigned to ABC subfamilies. Gene models were refined on the basis of sequence homology and EST support, and the subfamily assignment confirmed by protein BLAST analyses on the National Center for Biotechnology Information website. Using this strategy, 64 loci of putative *Daphnia *ABC genes were identified, for 48 of which evidence of mRNA expression was present in EST databases implemented in the JGI genome portal and wFeaBase (10,392 assembled cDNA on genome scaffolds) (Table 1). The majority of potential *Daphnia *ABC genes lacking EST support (14 of 16) were found in subfamilies G and H (Table 1), in which a high number of gene duplications was observed (see below for further discussion). Significant sequence homologies to the query NBDs were observed at further 15 loci that were excluded from further analysis [see additional file 1]. Of these loci, four corresponded to obvious pseudogenes, while two most likely represent bacterial contamination. At the remaining nine excluded loci, the best obtainable gene models were fragmentary, i.e. showing lack/incompleteness of vital domains, with many models being affected by sequence gaps. All excluded loci lacked EST evidence of expression.

**Table 1:** Characterisation of 64 *Daphnia pulex *ABC proteins.

**Subfamily**		**Protein ID**	**Location**	**Orientation**	**Size (amino acids)**	**Predicted topology**	**EST support ?**	**Comments**
**A**		346971	2:23668-15376	-	1719	(6TM-NBD)2	Y	

		312055	5:1495447-1485192	+	1818	(6/7TM-NBD)2	Y	

		312056	5:1496132–1505498	+	2147	(6TM-NBD)2	Y	

		347506	101:21050–30661	-	2199	(6TM-NBD)2	N	

								

**B**	full transporters	347265	8:420953–426161	-	1340	(5/6TM-NBD)2	N	

		347264	10:634775–640649	+	1293	(5/6TM-NBD)2	Y	

								

	half transporters	347270	9:1959479–1964061	-	701	6TM-NBD	Y	

		347266	23:432954–436338	-	688	3TM-NBD	Y	

		347268	30:67315–71521	-	835	11TM-NBD	Y	partial

		347275	98:485150–488520	+	713	4TM-NBD	Y	

		347276	213:109133–113467	-	661	5TM-NBD	Y	

								

**C**		347323	17:1390709–1397174	-	1191	5TM-6TM-6TM-NBD	Y	partial, affected by sequence gaps

		347281	74:386519–398940	-	1547	5TM-(5/6TM-NBD)2	Y	

		347292	75:395095–402681	+	1420	(6/3TM-NBD)2	Y	

		347295	75:403303–410701	+	1406	(6/4TM-NBD)2	Y	

		347288	133:190003–199540	-	1246	(3/6TM-NBD)2	Y	partial, affected by sequence gaps

		442500	133:231506–240432	+	1584	9TM-(6TM-NBD)2	Y	

		347548	444:25692–32505	-	1268	(6/3TM-NBD)2	Y	partial

								

**D**		347330	15:213474–218223	+	761	2TM-NBD	Y	

		347326	53:311868–315702	+	604	2TM-NBD	Y	

		303977	245:28669–31749	-	596	5TM-NBD	Y	

								

**E**		189585	173:120755–123775	+	610	NBD-NBD	Y	

								

**F**		304799	3:3598370–3601666	+	613	NBD-NBD	Y	

		347357	66:238375–242362	-	936	NBD-NBD	Y	

		347354	113:180264–184281	-	718	NBD-NBD	Y	

		347363	192:174461–177997	+	596	NBD-NBD	Y	

								

**G**		312940	7:1643106–1646831	-	628	NBD-7TM	N	

		347419	7:1650205–1654653	+	619	NBD-6TM	Y	

		312942	7:1655435–1658770	+	614	NBD-6TM	Y	

		222011	7:1676572–1680401	+	627	NBD-7TM	Y	

		312948	7:1681527–1684644	-	602	NBD-7TM	Y	

		312949	7:1684927–1688387	-	614	NBD-7TM	Y	

		312950	7:1689092–1692200	+	623	NBD-7TM	Y	

		312951	7:1692456–1696044	+	628	NBD-7TM	N	

		347409	12:762775–766127	-	699	NBD-5TM	N	

		314702	12:765851–769365	-	698	NBD-6TM	Y	

		347393	12:874106–877816	+	663	NBD-5TM	Y	

		315707	15:278488–281790	-	663	NBD-6TM	Y	

		347377	15:281541–286046	-	682	NBD-6TM	Y	

		347380	15:285548–289633	-	672	NBD-6TM	Y	

		320906	37:831802–835003	+	630	NBD-7TM	N	

		320907	37:834846–838359	-	617	NBD-6TM	N	

		347524	86:126420–129178	+	558	NBD-5TM	N	partial

		347412	86:129357–136390	+	637	NBD-6TM	N	

		327299	89:374068–377567	+	648	NBD-6TM	Y	

		258299	111:295149–303533	+	949	NBD-7TM	N	

		347416	131:1–10171	-	727	NBD-5TM	Y	partial, affected by sequence gaps

		347444	152:60036–65456	+	674	NBD-6TM	N	

		347523	1067:1352–4035	-	499	NBD-3TM	N	partial, affected by sequence gaps

								

**H**		46780	12:7469–11245	-	761	NBD-7TM	N	

		99394	12:12278–16254	-	781	NBD-6TM	Y	

		347465	12:36982–43559	-	766	NBD-5TM	Y	

		197573	30:1127689–1131913	+	767	NBD-5TM	Y	

		104532	30:1134028–1137924	+	739	NBD-6TM	N	

		197993	35:762822–771561	+	783	NBD-5TM	Y	

		347474	35:773437–778932	+	747	NBD-6TM	Y	

		347478	88:340140–344680	+	752	NBD-5TM	Y	

		201766	88:366247–371308	+	752	NBD-7TM	Y	

		347450	100:55123–59845	-	807	NBD-7TM	Y	

		328125	100:63078–67060	-	779	NBD-6TM	N	

		328127	100:72768–77501	-	793	NBD-7TM	Y	

		228828	121:311724–315411	-	738	NBD-5TM	Y	

		332183	182:193263–199213	-	801	NBD-6TM	Y	

		340396	4241:1–6396	+	481	NBD-1TM	N	partial, affected by sequence gaps

The column 'location' gives the scaffold number followed by the position coordinates of the gene model of the given protein ID in v1.1 of the *Daphnia pulex* draft genome sequence assembly [32].

To analyse the evolutionary position of the 64 putative *Daphnia *ABC transporters identified, phylogenetic analyses compared the complement of ABC proteins in the *Daphnia *genome to that in the genomes of yeast (*Saccharomyces cervisiae*), fruit fly (*Drosophila melanogaster*), worm (*Caenorhabditis elegans*) and human (*Homo sapiens*), using predicted (*Daphnia*) or database derived protein sequences [see additional file 2]. For two reasons, separate analyses were carried out for each subfamily. Firstly, the domain architecture of ABC transporters is highly variable among subfamilies, which complicates bioinformatic analyses on whole length sequences of the whole protein family. Secondly, while the NBDs of different transporters show a similar length and organisation, their sequence is too conserved to provide a meaningful degree of resolution in phylogenetic analysis.

### ABCA

**Figure 1:**
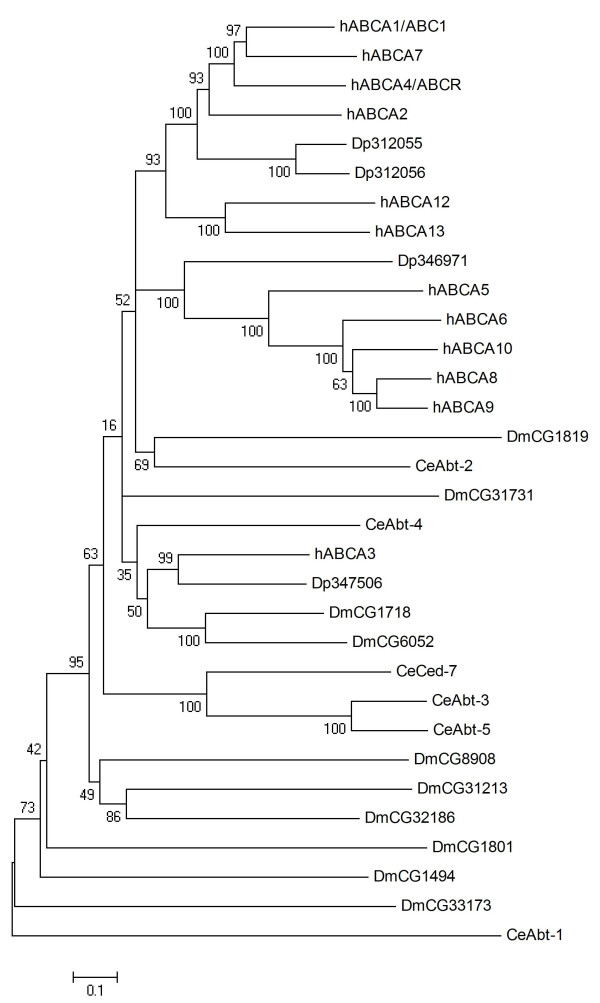
**Phylogenetic tree of ABCA proteins in four eukaryotic genomes**. Predicted amino acid sequences were aligned using ClustalX, and the alignment used to generate a phylogenetic tree using the neighbour joining method [74]. Bootstrapping was used to determine the relative support of the various branches (5,000 replicates, support expressed as percent) [74]. Dp, *Daphnia pulex*,; Dm *Drosophila melanogaster*; Ce *Caenorhabditis elegans*, h *Homo sapiens*; Sc *Saccharomyces cervisia*.

ABCA subfamily proteins are full transporters characterised by distinctive conserved traits, in particular a large extracellular loop between the first two transmembrane helices of each TMD, and a family specific motif located C-terminal of each NBF [33]. Four ABCA subfamily transporters presenting these hallmarks (data not shown) have been identified in *Daphnia *(Table 1). An analysis of the evolutionary relationship of these transporters to human, worm, and fruit fly ABCA proteins is shown in Fig. 1. Dappu-(*Daphnia pulex*)312055 and Dappu-312056 are neighbouring genes showing a head-to-tail orientation (Table 1) and display 59% amino acid identity, suggesting they are the result of a tandem duplication. Dappu-312055 and Dappu-312056 group together with hABCA1/ABC1, hABCA2, hABCA4/ABCR and hABCA7. Dappu-346971 groups together with the hABCA5 cluster (hABCA5, 6, 8, 9, and 10). Dappu-347506 groups together with hABCA3. In mammals, ABCA proteins adopt critical roles in the control of cellular lipid transport processes. Loss-of-function scenarios in human monogenetic diseases and mouse knockout models have revealed roles of hABCA1/ABC1 in HDL biogenesis, of hABCA3 in lung surfactant production, of hABCA4/ABCR in retinal integrity and of ABCA12 in keratinisation processes in the skin [34]. Thus, while ABCA proteins share a functional relation to lipid trafficking, individual transporters in this subfamily have adopted highly specialised roles in phospho- and sphingolipid export machineries [34]. In consequence, it is not possible to assign specific putative roles to the *Daphnia *ABCA transporters, though it appears likely that they are involved in lipid trafficking processes.

### ABCB

**Figure 2:**
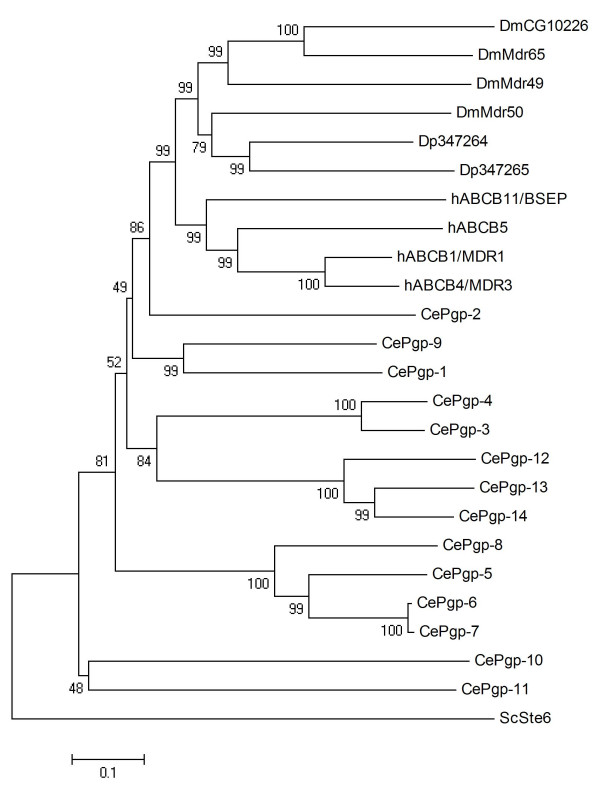
**Phylogenetic tree of ABCB subfamily full transporters in five eukaryotic genomes, derived according to the procedure outlined in the legend to Fig. 1**.

The ABCB family can be divided into a group of full transporters (FT) that includes the drug efflux pump hABCB1/MDR1, and a group of half transporters (HT). Two ABCB FTs and five ABCB HTs were found in the *Daphnia *genome (Table 1). The evolutionary analysis of ABCB FT assigned the human, worm, and arthropod (combined *Drosophila *and *Daphnia*) transporters into clearly distinguished clades (Fig. 2), suggesting that this subfamily has diversified through lineage-specific gene duplications. Early reports have noted the similarity in sequence of three *Drosophila *ABCB FTs and hABCB1/MDR1, and named these genes *mdr49*, *mdr50*, and *mdr65 *[35,36]. While no further information is available on *mdr50*, the *Drosophila *gene most closely related to the two *Daphnia *ABCB FTs, a number of studies suggests a role of *mdr49 *and to a lesser extent also *mdr65 *in the biochemical defence against toxicants. The disruption of the *mdr49 *gene resulted in an increase in colchicine resistance [35]. A genetic polymorphism related to α-amatin resistance in *Drosophila *was mapped to the region of the *mdr65 *gene [37]. Colchicine exposure and heat shock increases the expression of *mdr49*, but not *mdr65*, in *Drosophila *larvae, while both genes were induced in tumours [38]. *Mdr49 *was further found to be induced by polycyclic aromatic hydrocarbons, and shown to be involved in the transport of these chemicals [39].

**Figure 3:**
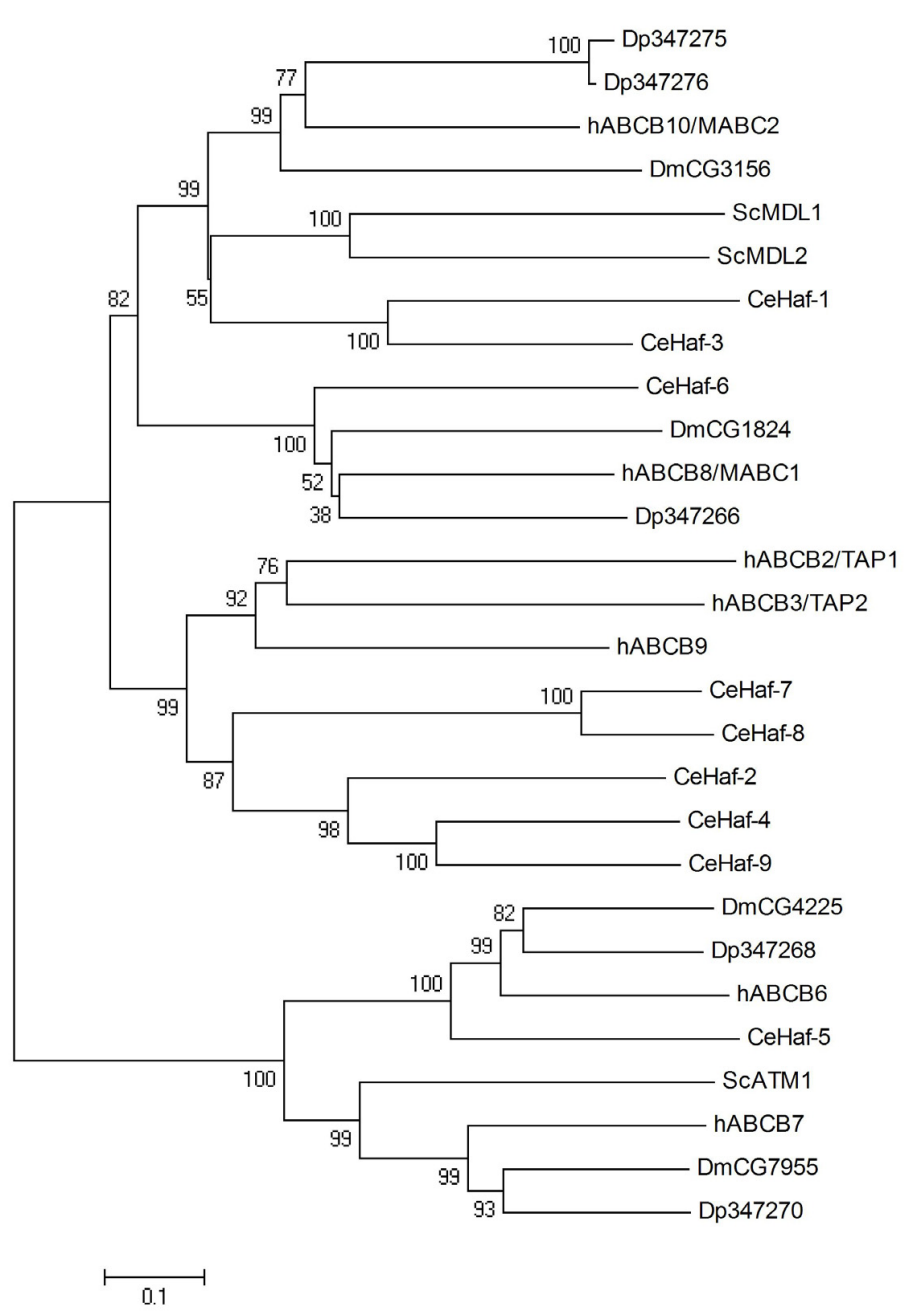
**Phylogenetic tree of ABCB subfamily half transporters in five eukaryotic genomes, derived according to the procedure outlined in the legend to Fig. 1**.

The phylogenetic analysis of ABCB HT revealed comparatively clear orthologue relations (Fig. 3). Human mitochondrial transporters hABCB6, hABCB7, hABCB8/MABC1 and hABCB10/MABC2 function in iron metabolism and transport of Fe/S protein precursors. The hABCB6, hABCB7, and hABCB8/MABC1 proteins each have one orthologue in *Daphnia *(Dappu-347268, Dappu-347270, and Dappu-347266, respectively). The hABCB10/MABC2 protein has two *Daphnia *co-orthologues, Dappu-347275 and Dappu-347276, which show high similarity (83% amino acid identity). The human transporter associated with antigen processing (TAP) is a heterodimer of two ABCB proteins, hABCB2/TAP1 and ABCB3/TAP2 [40]. TAP translocates peptides derived from proteasomal degradation from the cytosol to the lumen of the endoplasmic reticulum, where their loading onto major histocompatibility complex (MHC) class I molecules occurs [40]. The function of hABCB9/TAPL (TAP-like) is currently unknown and its subcellular localisation is still under discussion [41]. It has been shown that TAPL is present in the lysosomal compartment [42], and it has been recently suggested that it might be involved in peptide presentation to MHC class II in dendritic cells [41]. As invertebrates lack the mammalian adaptive immune response, the lack of TAP/TAPL homologues in *Daphnia *and *Drosophila *(Fig. 3) is not unexpected. However, the presence of proteins orthologous to TAP/TAPL in *C. elegans *(Fig. 3) suggests that these protein transporters might have further roles unrelated to antigen presentation.

### ABCC

**Figure 4:**
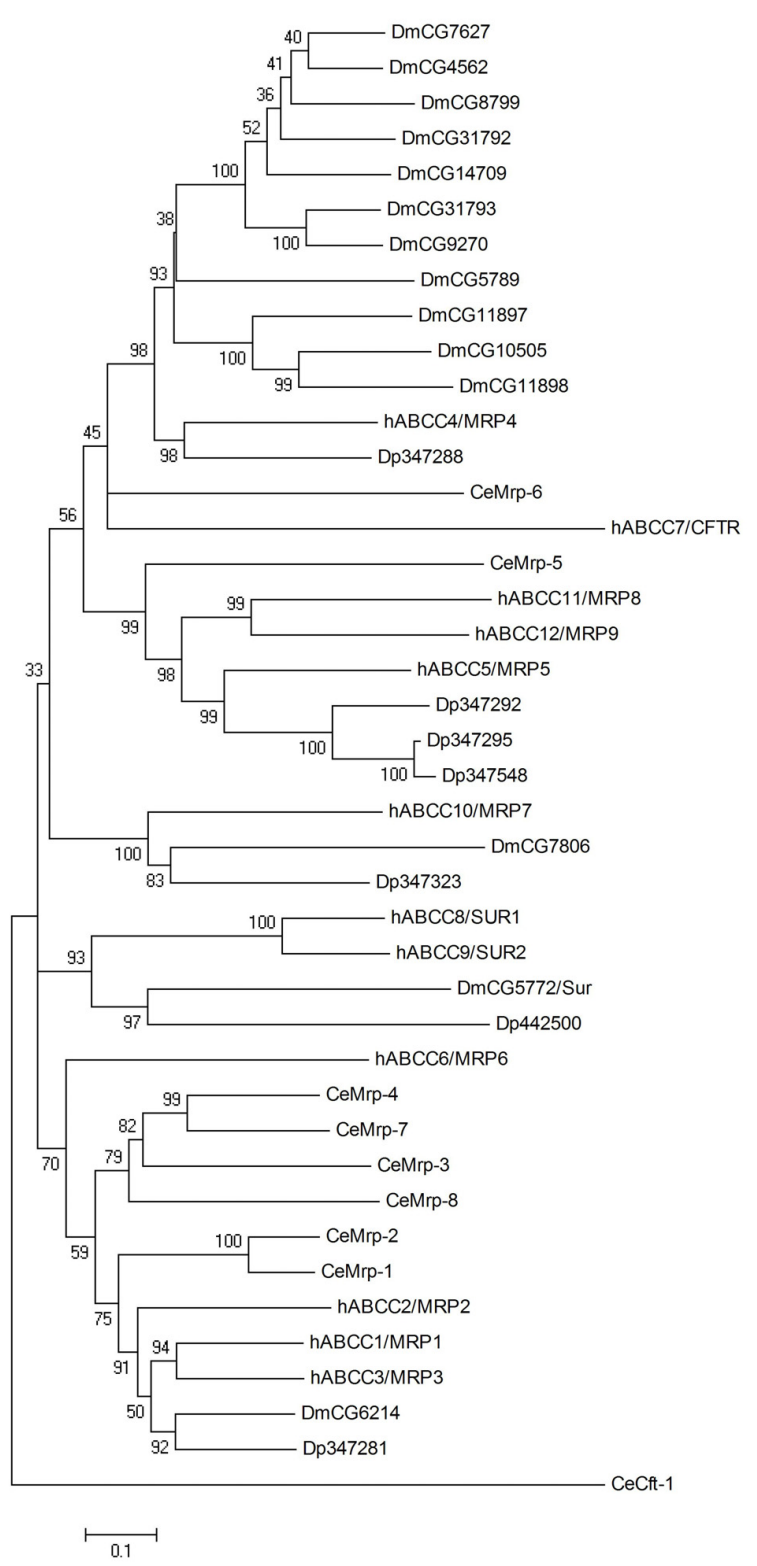
**Phylogenetci tree of ABCC proteins in five eukaryotic genomes, derived according to the procedure outlined in the legend to Fig. 1**.

The ABCC subfamily is functionally diverse, comprising the chloride channel CFTR (cystic fibrosis transmembrane conductance regulator), the membrane-bound receptors SURs (sulfonylurea receptors), and broad-specificity transporters called multidrug resistance-associated proteins (MRPs) that translocate a range of substrates including drugs, endogenous compounds and their glutathione and glucuronyl conjugates, glutathione, and cyclic nucleotides [43-45]. ABCC subfamily proteins are full-transporters showing two TMDs and two NBDs. Within the human ABCC family, hABCC8/SUR1, hABCC9/SUR2 and certain MRPs called 'long' MRPs are unique in that they possess an additional N-terminal TMD called TMD0 which is lacking in hABCC7/CFTR and the remaining 'short' MRPs [46,47]. Searches of the *Daphnia *genome identified seven ABCC transporters (Table 1). The phylogenetic tree of *Daphnia*, human, worm, and *Drosophila *ABCC proteins is characterised by comparatively low support of the more basal nodes, most probably reflecting the heterogeneity of the ABCC subfamily (Fig 4). Dappu-442500 is found in the same, well supported clade as human and *Drosophila *SURs, suggesting it is a SUR homologue. Indeed, Dappu-442500 displays a general architecture of SUR and 'long' MRPs, possessing the additional N-terminal TMD0 (Table 1). Moreover, Dappu-442500 shows a significant conservation of two SUR typical N-terminal motifs, the sulphonylurea receptor family signature (PR01092) and the sulphonylurea receptor type 1 family signature (PR01093), with predicted amino acid similarities to hABCC8/SUR1 of 73% and 61%, respectively (data not shown). Together with previous reports of SUR-typical functional traits of the *Drosophila *protein *CG5772 *[48,49], the data strongly suggests that Dappu-442500 is indeed a SUR. Further *Daphnia *ABCC proteins possessing an additional N-terminal TMD0 are Dappu-347281 and Dappu-347323 (Table 1). In our phylogenetic analysis Dappu-347281 groups together with *Drosophila CG6214 *(Fig 4), which is a long MRP resembling hABCC1/MRP1 but awaiting in-depth functional characterisation [50,51]. According to the tree obtained in this study, Dappu-347323 is a putative orthologue of hABCC10/MRP7 and *Drosophila CG7806 *(Fig. 4). hABCC10/MRP7 is capable of conferring a hABCC1/MRP1-type multidrug resistance phenotype in cellular models, but its physiological function is at present poorly understood [52]. Functional data are lacking on *CG7806*. The remaining *Daphnia *MRPs, Dappu-347288, Dappu-347292, Dappu-347295, and Dappu-347548, show the structural traits of 'short' MRPs (Table 1). The hABCC4/MRP4 protein groups together with Dp347288 in a clade of moderately low bootstrap support (Fig. 4) and is the ABCC transporter showing the greatest similarity to the *Daphnia *protein (42% amino acid identity). Dappu-347292, Dappu-347295, and Dappu-347548 group together with hABCC5/MRP5 in a clade of high bootstrap support (Fig. 4). The hABCC4/MRP4 and hABCC5/MRP5 proteins have been reported to be able to transport cyclic nucleotides *in vitro*, but the *in vivo *relevance of this observation is controversial and their physiological roles remain to be unravelled [53]. Dappu-347288 groups together in one clade with hABCC4/MRP4 and a cluster of *Drosophila *ABCC proteins including *CG10505 *(Fig. 4). *CG10505 *is regulated by heavy metals via the metal-responsive transcription factor 1, and has been shown to be involved in biochemical detoxification of zinc and copper [54]. This parallels previous studies showing that cell lines overexpressing hABCC1/MRP1 are resistant to arsenite and antimony (reviewed in [47]). Together these data suggest *Daphnia *MRPs could represent potential biochemical factors in the defence against toxicants; however, in the absence of functional data and in the view of the complex phylogeny of the ABCC subfamily this remains at present speculative.

### ABCD

**Figure 5:**
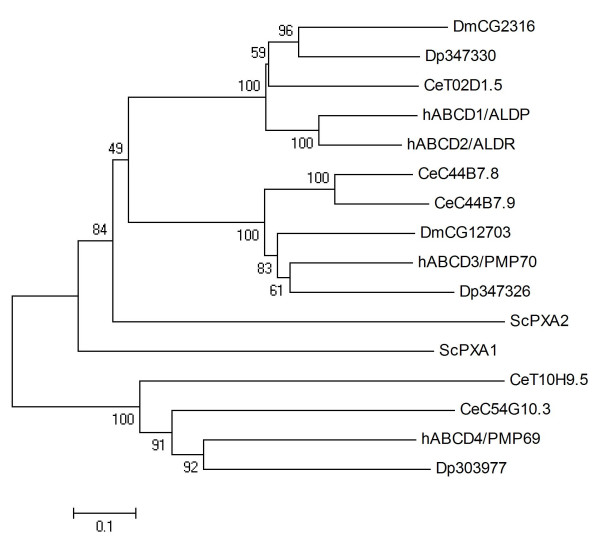
**Phylogenetic tree of ABCD proteins in five eukaryotic genomes, derived according to the procedure outlined in the legend to Fig. 1**.

The ABCD subfamily contains half transporters located to the peroxisome that are involved in the import of fatty acids and/or fatty acyl-CoAs into this organelle [55]. The simultaneous posttranscriptional silencing of three *C. elegans *ABCD transporters disrupted offspring production in a previous study, suggesting developmental roles of peroxisomal ABC transporters [56]. Mutations in the hABCD1/ALDP gene are the principal inherited defect in adrenoleukodystrophy, a clinically heterogeneous X-linked recessive disorder characterised by adrenal insufficiency and neuronal demyelination [57]. This study identified three ABCD transporters in the *Daphnia *genome, Dappu-347330, Dappu-347326, and Dappu-303977 (Table 1). The phylogenetic analysis revealed that three clades of ABCDs exist in metazoans, each of which has one *Daphnia *member (Fig 5). The high degree of conservation (amino acid identity of *Daphnia *ABCD proteins to the closest human homologue between 46% and 55%) and the clear structure of the phylogenetic tree are consistent with the notion that the function of ABCD proteins in *Daphnia *might resemble that in other metazoans.

### ABCE and ABCF

**Figure 6:**
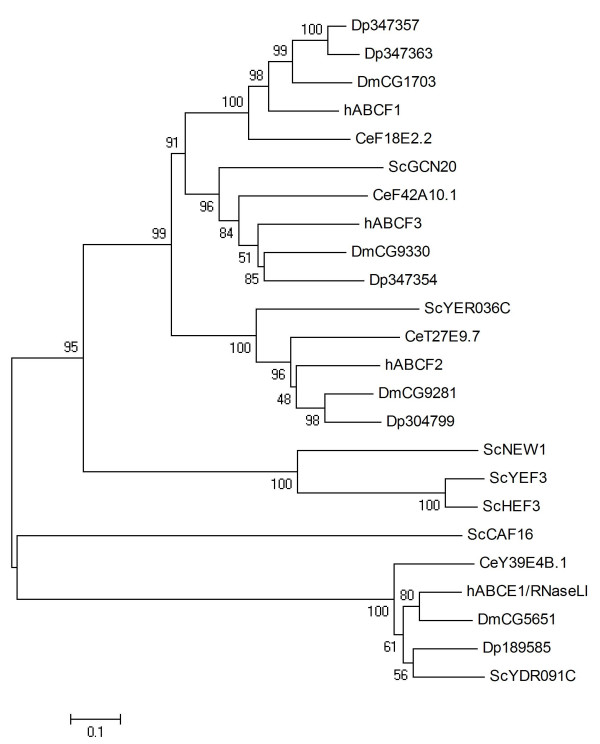
**Phylogenetic tree of ABCE and ABCF proteins in five eukaryotic genomes, derived according to the procedure outlined in the legend to Fig. 1**.

The subfamilies ABCE and ABCF are comprised of atypical ABC proteins that contain a pair of linked nucleotide binding domains and lack transmembrane domains [58]. The proteins of both subfamilies are highly conserved across evolutionary diverse taxa, suggesting their role in fundamental cell biological processes. Most eukaryotes possess one ABCE protein, and *Daphnia *conforms to this rule (Dappu-189585, Table 1). Human ABCE1/RNaseLI was initially identified as an inhibitor of RNase L [59]. Recent data indicate that human and yeast ABCE proteins have further a central role in translation initiation [60]. ABCF proteins have roles in ribosome assembly and/or protein translation [13]. Four ABCF proteins have been identified in *Daphnia *(Table 1). The phylogenetic analysis of ABCE and ABCF proteins was carried out together (Fig. 6). As expected, Dappu-189585 fell into the clade containing other ABCE proteins. ABCF proteins were divided into four well-supported clades, one of which contained only yeast proteins, while the remaining three each contained one of the human ABCFs (Fig. 6). Of the *Daphnia *ABCF proteins, of which Dappu-347357 and Dappu-347363 show 53% amino acid identity and are homologues to hABCF1, while Dappu-304799 and Dappu-347354 are homologues to hABCF2 and hABCF3, respectively.

### ABCG

As half transporters, ABCG proteins need to form homo- or hetero dimers to form a functional pump. In contrast to other ABC half transporters, ABCG proteins show a reverse domain architecture, with the TMD being located C-terminally of the NBD. In human, hABCG1/WHITE1 and hABCG4 are involved in cellular cholesterol efflux to high density lipoprotein, hABCG5 and hABCG8 mediate the intestinal and biliary efflux of cholesterol, plant, and shellfish sterols, while hABCG2/BCRP is a drug efflux pump [61]. The most intensively studied ABCG protein is the *white *protein in *Drosophila*, which dimerises with ABCG proteins *brown *and *scarlet *to function as a transporter of eye pigment precursors [62]. In yeast and in plants, certain ABCG subfamily proteins are reverse full transporters and called PDR (pleiotropic drug resistance) proteins [63]. Because of their duplicated domain structure (NBD-TM-NBD-TM), yeast PDRs were not included in the phylogenetic analyses of this study.

**Figure 7:**
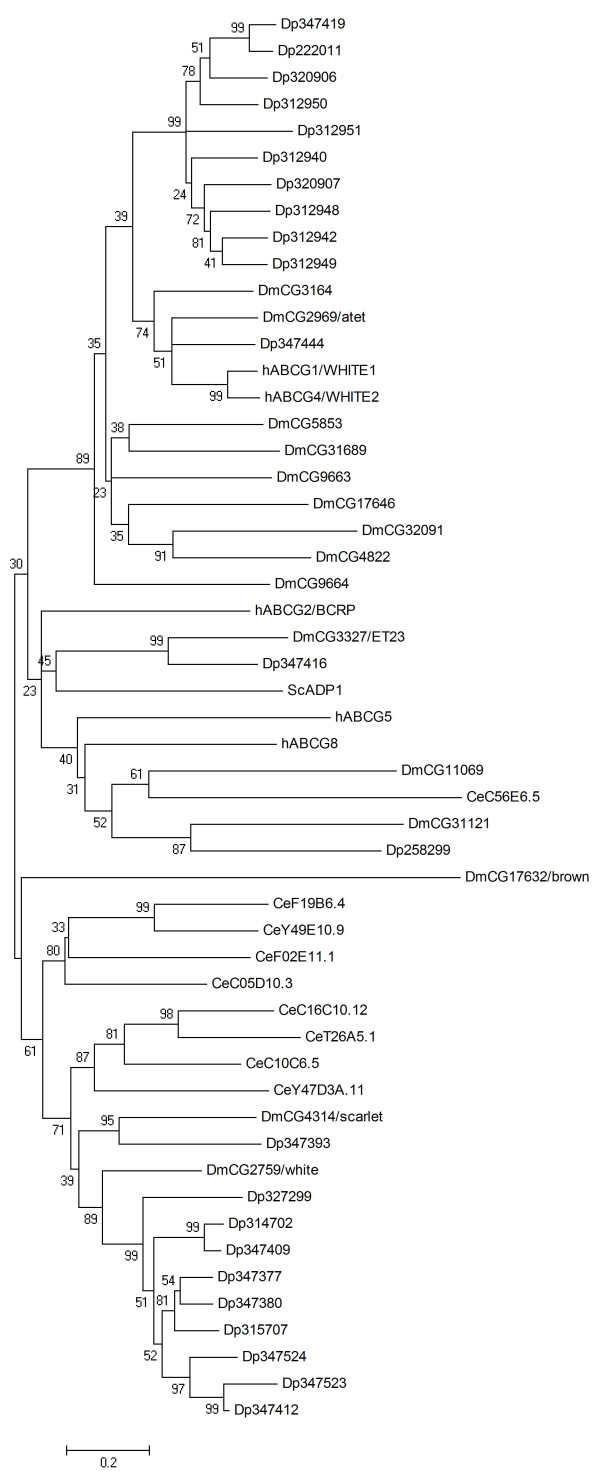
**Phylogenetic tree of ABCG proteins in five eukaryotic genomes, derived according to the procedure outlined in the legend to Fig. 1**.

The identification of *Daphnia *ABC transporters revealed that in this species ABCG proteins form the largest ABC subfamily (23 members, Table 1). This parallels the situation in *Drosophila *[2]. The phylogenetic analysis of the ABCG subfamily revealed that the high number of ABCG genes in *Daphnia *and *Drosophila *is due to extensive lineage specific gene duplications (Fig. 7). It is know that the genomes of flies and worms contain a large number of duplicated genes, with a greater number of tandem or locally duplicated genes in the *C. elegans *than the *Drosophila *genome [64]. A large number of *C. elegans *annotated genes might be pseudogenes [65,66]. A similar situation seems to exist in *Daphnia*, at least with respect to the ABCG (and ABCH, see next section) family, which comprises several clusters of putative genes located in close vicinity displaying high predicted amino acid sequence similarity (up to 86%), and which further show a high number of loci lacking EST support (10 of 23 ABCG proteins Table 1). It seems premature, however, to conclude that the non-expressed putative ABC gene loci (Table 1) are pseudogenes. For instance, a detailed functional analysis of tandem duplicated ABC genes in the worm has revealed differential function of the duplicates, with expression being highly tissue or stage specific [67].

In the phylogenetic analysis of ABCG proteins from the *Daphnia *and other genomes, Dappu-347419 and nine further *Daphnia *proteins forms a clade (Fig 7, top of tree) that consists of a cluster of eight neighbouring genes on scaffold 7 and two neighbouring genes on scaffold 37 (Table 1). Further down, Dappu-347444 groups together with *Drosophila *proteins *CG3164 *and *atet *(ABC transporter expressed in trachea, [68]) as well as hABCG1 and 4. Dappu-347416 is a homologue of the *Drosophila ET23 *gene product protein *CG3327*, an ABCG transporter believed to regulate intracellular ecdysone concentrations during development [69]. Phylogenetic analyses grouped Dappu-258299 together in one clade with hABCG5 and 8, and two *Drosophila *proteins of unknown function. Among the *Drosophila *eye pigment transporters, the protein *brown *(*CG17632*) does not have a *Daphnia *orthologue, Dappu-347393 is an orthologue of the *scarlet *protein (*CG4314*), while the *Drosophila white *protein has nine co-orthologues in *Daphnia*. Assuming that these proteins might adopt roles in eye pigment transport in *Daphnia *appears a reasonable hypothesis. However, it is noteworthy that recent evidence in *Drosophila *suggests additional neurobiological functions for the *white *protein [70,71].

### ABCH

**Figure 8:**
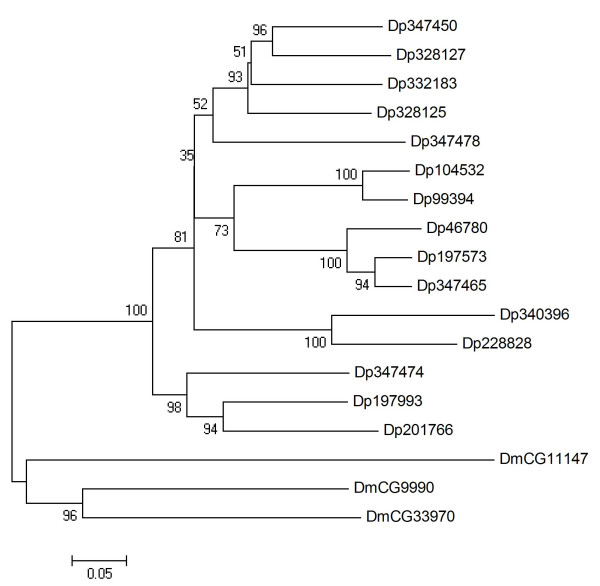
**Phylogenetic tree of ABCH proteins in five eukaryotic genomes, derived according to the procedure outlined in the legend to Fig. 1**.

The ABCH subfamily is lacking members in mammals and *C. elegans*, and has been identified for the first time in *Drosophila *[2]. At present, teleost fish are the only vertebrates known to possess ABCH transporters [6,8]. ABCH proteins are inverse half-transporters showing the same domain architecture as the members of the ABCG subfamily. The function of ABCH proteins is yet unknown. In *Daphnia*, this subfamily is with 15 members the second largest ABC subfamily (Table 1). The phylogenetic analysis assigned *Daphnia *and *Drosophila *proteins in distinct clades (Fig. 8), suggesting that the diversity of ABCH subfamily in *Daphnia *has arisen from lineage specific gene duplications. As observed with the ABCG subfamily, clusters of neighbouring genes displaying high similarity are found among *Daphnia *ABCH proteins (Table 1).

## Conclusion

The identification and phylogenetical analysis of ABC transporters in the *Daphnia pulex *genome, the first available crustacean genome, has revealed interesting traits of this important group of proteins in *Daphnia*. The complement of ABC proteins shows parallels between two arthropods *Daphnia *and *Drosophila *in that both lack homologues to the human proteins TAP, protein translocators involved in antigen processing, and CFTR, a chloride channel regulated by ATP. The high conservation in *Daphnia *of ABC proteins involved in fundamental cellular processes, such as the mitochondrial ABCB half transporters and the members of subfamilies ABCD, ABCE and ABCF, confirms an earlier study [21] in strongly suggesting an evolutionary ancestral origin of these proteins. Clear orthologous relationships were observed in further occasions, providing the basis for a tentative function assignment to *Daphnia *homologues of SUR, a membrane receptor regulating a potassium channel, and to *Daphnia *homologues of the *Drosophila *genes *ET23 *and *scarlet*. The presence of proteins in *Daphnia *that resemble the ABC drug efflux transporters hABCB1/MDR1 and hABCC1/MRP1 is in accordance with the hypothesis that these *Daphnia *proteins might adopt roles in the biochemical defence against toxicants; however, the lack of clear orthologous relationships makes the inference of function in these cases uncertain. A high number of duplicated genes was observed in the ABCG and ABCH subfamilies of *Daphnia*. This parallels the situation in *Drosophila *and *Caenorhabditis*, which also show lineage specific duplications, albeit in other subfamilies. More research is needed to unravel the physiological significance of gene duplications in *Daphnia*.

## Methods

To identify loci of genes encoding ABC transporters, tblastn searches were performed on v1.1 (September, 2007) of the *Daphnia pulex *draft genome sequence assembly [32] using the highly conserved NBD [72] of different *Drosophila melanogaster *ABC proteins as queries. One search was carried out per subfamily, each using the sequence of the NBD (as defined by interpro domain IPR003439) of a representative *Drosophila *protein (A: *CG1718*; B: *CG3879 (mdr49)*; C: *CG9270*; D: *CG12703*; E: *CG5651*; F: *CG9330*; G: *white*; H: *CG9990*). If the *Drosophila *transporter had two NBDs, the N-terminal domain was used. Hits from individual subfamily specific tblastn searches (E-value of 10^-5^) significantly overlapped, with each search retrieving loci of genes of the query and other subfamilies. Increasing the E-value to 10^-4 ^increased the degree of overlap between individual searches, but had no effect of the total number of loci retrieved, suggesting that our search strategy gave an exhaustive representation of those sequences in the current version of the *Daphnia pulex *genome that show homology to ABC-transporter NBD domains. For each locus identified in tblastn searches, one preliminary gene model representing the NBD hit with reasonable fidelity was chosen from the gene models automatically generated by the JGI pipeline. To assign putative *Daphnia *ABC genes to ABC subfamilies, the NBDs of preliminary gene models were extracted using the ScanProsite facility [73] with predicted protein sequences and the prosite profile PS50893. NBDs were then subjected to a phylogenetic analysis together with those of fruit fly and human ABC transporters, using neighbour joining and bootstrapping with 5000 replicates in the program package MEGA4 [74]. NBFs of the fruit fly and human ABC transporters formed subfamily specific clusters, with separate groupings for the N- and C-terminal NBFs of full transporters (data not shown). This allowed unequivocal assignment of *Daphnia *transporters to ABC subfamilies. Gene models were then refined on the basis of homology and EST support. Both the tandem architecture of ABC transporters of certain subfamilies and the commonness of tandem gene duplications in *Daphnia *made manual adjustments to the gene models necessary, as machine generated gene models would sometimes combine exons from distinct genes. The subfamily assignment of *Daphnia *ABC proteins was confirmed by protein BLAST analyses of the manually corrected models on the National Center for Biotechnology Information website. Separate phylogenetic analyses on *Daphnia*, yeast, worm, fruitfly and human ABC transporters were then carried out per subfamily, using the same methodology as above [74] on full sequences.

## Authors' contributions

AS identified and annotated ABC proteins in *Daphnia *and wrote the manuscript. PC facilitated the bioinformatic analyses, particularly with respect to accessing and extracting database information and domain identification. MD contributed expert knowledge on ABC transporters and phylogenetic analysis, took part in the revision of this article. All authors read and approved the final manuscript.

## Supplementary Material

Additional File 1 **Table S1**. Loci that were identified in a tBLASTn search of the *Daphnia pulex *genome (see Methods) but not considered further because likely representing pseudogenes (1), or consisting of short partial sequences (2), or being likely bacterial contamination (3). Click here for file

Additional File 2 **Table S2**. Genbank accession numbers of yeast, worm, fruit fly and human ABC transporter sequences used in the phylogenetic analyses. Click here for file
