# Supplementary material for: The ABC transporter gene family of Daphnia pulex
Source: BMC Genomics. 2009 Apr 21;10:170. doi: 10.1186/1471-2164-10-170 (PMC2680897; doi:10.1186/1471-2164-10-170)
Supplement: Additional File 1 — Table S1. Loci that were identified in a tBLASTn search of the Daphnia pulex genome (see Methods) but not considered further because likely representing pseudogenes (1), or consisting of short partial sequences (2), or being likely bacterial contamination (3). [file 1471-2164-10-170-S1.doc]

Supplementary table 1. Loci that were identified in a tBLASTn search of the *Daphnia pulex* genome (see Methods) but not considered further because likely representing pseudogenes (1), or consisting of short partial sequences (2), or being likely bacterial contamination (3).

| **Protein ID** | **Position** | **Orientation** | **Sub-family** | **Size  (amino acids)** | **Predicted topology** | **Model impacted by sequence gaps?** | **Best BLAST hit** |
| --- | --- | --- | --- | --- | --- | --- | --- |
| *1) Loci with clear signs of pseudogenisation (exons with internal stop codons)* | | | | | | | |
| 328283 | 99:37705-42097 | - | C | 726 | NBD-5TM-NBD |  | PREDICTED: similar to ATP-binding cassette, sub-family C, member 5 isoform 1 isoform 2 [Macaca mulatta] XP_001104746 |
| 38461 | 14166:1-1035 | - | C | 300 | 2TM-NBD | Y | hypothetical protein [Yarrowia lipolytica] XP_503935 |
| 327857 | 96:129027-133437 | + | F | 288 | NBD |  | ATP-binding cassette sub-family F member 1. [Sus scrofa] Q767L0 |
| 247555 | 40:632500-635285 | + | F | 188 | NBD |  | ENSANGP00000000043 [Anopheles gambiae str. PEST] XP_306294 |
| *2) Partial sequences lacking vital parts of a functional ABC transporter* | | | | | | | |
| 68106 | 1417:7858-8877 | - | B | 220 | NBD | Y | CG4225-PA [Drosophila melanogaster] NP_650503 |
| 65310 | 213:133438-133719 | + | B | 78 | NBD |  | ENSANGP00000002692 [Anopheles gambiae str. PEST] XP_312209 |
| 442567 | 60:287404-291248 | + | G | 451 | NBD-TM | Y | PREDICTED: similar to CG3327-PA, isoform A [Tribolium castaneum] XP_975214 |
| 8579 | 147:9765-10001 | + | G | 65 | NBD |  | Protein white. [Lucilia cuprina] Q05360 |
| 16735 | 189:112876-113126 | - | G | 69 | NBD |  | Protein white. [Lucilia cuprina] Q05360 |
| 336951 | 1261:1-971 | - | H | 148 | NBD | Y | PREDICTED: similar to CG9990-PA, isoform A [Tribolium castaneum] XP_974932 |
| 342335 | 7171:1-1684 | + | H | 275 | NBD-1TM | Y | PREDICTED: similar to CG9990-PA, isoform A isoform 1 [Apis mellifera] XP_393164 |
| 17514 | 2709:6255-6696 | - | H | 122 | NBD | Y | PREDICTED: similar to CG9990-PA, isoform A [Tribolium castaneum] XP_974932 |
| 337061 | 1321:9018-9644 | + | H | 152 | NBD | Y | PREDICTED: similar to CG9990-PA, isoform A isoform 1 [Apis mellifera] XP_393164 |
| *3) Sequences that are likely bacterial contamination* | | | | | | | |
| 347511 | 29:528902-530125 | + | none | 261 | 3TM-NBD | Y | inner-membrane translocator [Acidovorax avenae subsp. citrulli AAC00-1] YP_970604 |
| 66764 | 461:71873-73073 | - | none | 331 | NBD | Y | peptide ABC transporter, ATP-binding protein [Verminephrobacter eiseniae EF01-2] ZP_01400776 |
