# Supplementary material for: The ABC transporter gene family of Daphnia pulex
Source: BMC Genomics. 2009 Apr 21;10:170. doi: 10.1186/1471-2164-10-170 (PMC2680897; doi:10.1186/1471-2164-10-170)
Supplement: Additional File 2 — Table S2. Genbank accession numbers of yeast, worm, fruit fly and human ABC transporter sequences used in the phylogenetic analyses. [file 1471-2164-10-170-S2.doc]

Supplementary table 2. Genbank accession numbers of yeast, worm, fruit fly and human ABC transporter sequences used in the phylogenetic analyses.

| ABC subfamily | *S. cervisiae* | *C.elegans* | *D. melanogaster* | *H. sapiens* |
| --- | --- | --- | --- | --- |
| A | - | Abt-1 CAA18775  Abt-2 NP_490949  Abt-3 CAB05222  Abt4 AAC69223  Abt-5 CAA22142  Abt-6 AAB54203  Ced-7 NP_499115 | CG1494 AAF50838  CG1718 AAF50837  CG1801 AAF50836  CG1819 AAF50847  CG31213 NP_732473  CG31731 NP_723838  CG32186 NP_730301  CG33173 NP_788912  CG6052 AAF49312  CG8908 AAF57490 | hABCA1/ABC1 NP_005493  hABCA2 NP_001597  hABCA3 CAA65825  hABCA4/ABCR AF001945  hABCA5 NP_061142  hABCA6 NP_525023  hABCA7 AF328787  hABCA8 AB020629  hABCA9 NP_525022  hABCA10 XP_085647  hABCA12 NP_056472  hABCA13 NP_689914 |
| B – full transporters | Ste6 CAA82054 | Pgp-1 CAB01232  Pgp-2 AAB52482  Pgp-3 CAA91467  Pgp-4 CAA91463  Pgp-5 CAA94202  Pgp-6 CAA94220  Pgp-7 CAA94219  Pgp-8 CAA94203  Pgp-9 CAB03973  Pgp-10 AAC48149  Pgp-11 CAA88940  Pgp-12 CAA91799  Pgp-13 CAA91800  Pgp-14 CAA91801 | CG10226 AAF50670  Mdr49 NP_523724  Mdr50 NP_523740  Mdr65 NP_476831 | hABCB1/MDR1 4505769  hABCB4/MDR3 AAA36207  hABCB5 AAO73470  hABCB11/BSEP AF091582 |
| B – half transporters | MDL1 NP_013289  MDL2 NP_015053  ATM1 NP_014030 | Haf-1 CAB02812  Haf-2 AAC71121  Haf-3 CAB09418  Haf-4 AAC68724  Haf-5 CAB04947  Haf-6 AAK29911  Haf-7 CAB60586  Haf-8 CAB16503  Haf-9 AAK39394 | CG1824 AAF48177  CG3156 NP_569844  CG4225 AAF55241  CG7955 AAF47525 | hABCB2/TAP1 CAA40741  hABCB3/TAP2 AAA59841  hABCB6 NP_005680  hABCB7 AB005289  hABCB8/MABC1 AF047690  hABCB9 AC002486  hABCB10/MABC2 XP_001871 |
| C | YCF1 NP_010419  BPT1 NP_013086  YBT1 NP_013052  YOR1 NP_011797  YHL035C NP_011828  YKR103W NP_013029 | Cft-1 AAK52175  Mrp-1 AAD31550  Mrp-2 AAB07022  Mrp-3 CAA92148  Mrp-4 CAB02667  Mrp-5 CAB54225  Mrp-6 AAA82317  Mrp-7 CAA21622  Mrp-8 CAA22110 | CG10505 AAF46706  CG11897 AAF56869  CG11898 AAF56870  CG14709 AAF54656  CG31792 NP_724148  CG31793 NP_609930  CG4562 AAF55707  CG5789 AAF56312  CG6214 AAF53223  CG7627 AAF52648  CG7806 AAF52639  CG8799 AAF58947  CG9270 AAF53950  Sur NP_477472 | hABCC1/MRP1 AAB46616  hABCC2/MRP2 CAA65259  hABCC3/MRP3 AB010887  hABCC4/MRP4 NP_005836  hABCC5/MRP5 AAB71758  hABCC6/MRP6 AF076622  hABCC7/CFTR AAC13657  hABCC8/SUR1 AAB02278  hABCC9/SUR2 AF061323  hABCC10/MRP7 NP_258261  hABCC11/MRP8 NP_149163  hABCC12/MRP9 NM_033226 |
| D | PXA1 NP_015178  PXA2 NP_012733 | C44B7.8 AAA68339  C44B7.9 AAA68340  C54G10.3 CAA99810  T02D1.5 CAB0590  T10H9.5 AAC19238 | CG12703 AAF49018  CG2316 AAF59367 | hABCD1/ALDP CAA79922  hABCD2/ALDR NP_005155  hABCD3/PMP70 CAA41416  hABCD4/PMP69 AF009746 |
| E | CAF16 NP_116625  YDR091C NP_010376 | Y39E4B.1 CAB54424 | CG5651 AAF50342 | hABCE1/RNase LI CAA53972 |
| F | GCN20 NP_116664  YER036C NP_010953  NEW1 NP_015098  YEF3 NP_013350  HEF3 NP_014384  YDR061W NP_010346 | F18E2.2 CAA99835  F42A10.1 AAA19072  T27E9.7 CAB04880 | CG1703 AAF48069  CG9281 AAF48493  CG9330 AAF49142 | hABCF1 AAH34488  hABCF2 NP_005683  hABCF3 NP_060828 |
| G | ADP1 NP_009937  PDR5 NP_014796  PDR10 NP_014973  PDR11 NP_012252  PDR12 NP_015267  PDR15 NP_010694  YOL075C NP_014567  SNQ2 NP_010294  YOR011W NP_878167  YNR070W NP_014468 | C05D10.3 AAA20989  C10C6.5 CAB05682  C16C10.12 CAA86750  F02E11.1 AAB66050  F19B6.4 CAA93461  T26A5.1 AAC77504  Y47D3A.11 CAB57891  Y49E10.9 CAB11549  C56E6.5 AAA81094 | Atet AAF51027  Brown AAF47020  CG11069 AAF56361  CG17646 AAF51341  CG31121 NP_733058  CG3164 AAF51548  CG31689 NP_722827  CG32091 NP_729728  CG3327 AAF51122  CG4822 AAF51552  CG5853 AAF52835  CG9663 AAF51130  CG9664 AAF51131  Scarlet AAF49455  White AAF45826 | hABCG1/WHITE1 AAC51098  hABCG2/BCRP XP_032425  hABCG4/WHITE2 NP_071452  hABCG5 AF320293  hABCG8 AF320294 |
| H | - | - | CG11147 AAF52284  CG33970 NP_001034071  CG9990 AAF56807 | - |
